# Effect of experimental hyperthyroidism on *CatSper1* and *CatSper2* genes expression in the seminiferous tubules of BALB/c mice: An experimental study

**DOI:** 10.18502/ijrm.v13i8.7501

**Published:** 2020-08-19

**Authors:** Saeed Sadeghi, Mahdi Jalali, Mohamad Reza Nikravesh, Mojtaba Sankian

**Affiliations:** ^1^Department of Anatomy and Cell Biology, School of Medicine, Mashhad University of Medical Science, Mashhad, Iran.; ^2^Immunology Research Center, School of Medicine, Mashhad University of Medical Science, Mashhad, Iran.

**Keywords:** CATSPER1, CATSPER2, Hyperthyroidism, Mice, Sperm.

## Abstract

**Background:**

*CATSPER 1* (Cation Channel Sperm Associated 1) and *CATSPER2* channels have an important role in sperm motility. In this study, the effects of hyperthyroidism on *Catsper1* and *2* genes of seminiferous tubules in mice testes were investigated.

**Objective:**

The present study was conducted to investigate the effect of hyperthyroidism on the expression of *CATSPER1* and *CATSPER2* genes in the seminiferous tubules of mice.

**Materials and Methods:**

This study was conducted on 20 BALB/C male mice divided into two groups - experimental and control. The experimental group was administered with 500 mg/l levothyroxine (L-thyroxine) liquid solution for two months for inducing hyperthyroidism, which was confirmed by radioimmunoassay. On the other hand, the control group was kept in animal houses under a normal condition. The implementation of real-time polymerase chain reaction and immunohistochemical studies was accomplished after the removal of the testes of the mice under anesthesia induced by chloroform.

**Results:**

Results showed that there was no significant difference in *CATSPER1* (p = 0.45) and *CATSPER2* (p = 0.34) gene expression between groups. At the same time, the color intensity showed no significant enhancement in the hyperthyroidism group (*CATSPER1* p = 0.17 and *CATSPER2* p = 0.22) as compared to the control group.

**Conclusion:**

Considering the key role of *CATSPER* in the molecular structure of the sperm, our findings showed that the hyperactivity of the thyroid gland has no significant effects on the function of these components. Therefore, it might be concluded that hyperthyroidism has no considerable effects on the seminiferous tubules.

## 1. Introduction

The endocrine system is the second most important regulatory system in the body after the nervous system. Thyroid-stimulating hormone (THS) stimulates the thyroid gland to secrete thyroxin, and then triiodothyronine hormones. Although THS plays different roles in many cells and tissues of the body, the function of this hormone in sperm development requires further research (1). Thyroid hormone activates the function of a wide range of genes that guide nuclear receptors through complicated signaling pathways (2). The testicles are mainly composed of seminiferous tubules supported by the interstitial connective tissue. Each tubule consists of three layers, namely basement membrane, myoid cells, and elastic fibers. Sertoli cells in BM play multiple roles, including the establishment of a blood-testis barrier (3). Excessive thyroid hormones cause hyperthyroidism, which is associated with decreased testicular mass, testosterone level, and Sertoli cells (4, 5). Thyroid hormone receptors (TR) are located on Sertoli cells. Different patterns of TR gene expression include TRα and TRβ, which are the main factors in the regulation of the effect of the thyroid hormones on the testes (6-8).

Mammalian fertilization is one of the most amazing biological phenomena, involving the infusion of the sperm with the oocyte. Calcium channels play an important role in various mammalian sperm functions, such as capacitation and acrosome reaction (9, 10). The cation channel of sperm (*CATSPER*) is a type of voltage-sensitive ion channel that allows the entrance of calcium ions. Sperm motility is the most important factor in fertilization, which is mainly regulated by calcium signaling (11, 12). *CATSPER1* is found in the tail of the sperm, and *CATSPER2* is required for the entrance of calcium ions to the sperm (13-15). The *CATSPER* gene encodes a six-transmembrane-spanning repeat that its product are only expressed in testes. *CATSPER* gene produces the four repeat structure of voltage-dependent Ca${}^{+2}$-chanel and cyclic-nucleotide-gated before duplication. Computer-assisted sperm analysis approved that every mutant of *CATSPER* gene reduced sperm motility and male fertility function for sperm penetration of the zona pellucida in mice. In addition, investigation showed that hyperactivated motility needs a sperm-specific pH-gated calcium channel in mice. So that increasing intracellular pH induced a rise in intracellular calcium. The amount of calcium regulate sperm motility through the motor proteins of the tail sperm (16, 17).

According to the importance of cation channel (*CATSPER1* and *CATSPER2*) in motility and the function of sperm, the aim of the present study was to investigate the effect of hyperthyroidism on the expressions of *CATSPER1* and *CATSPER2* genes in the seminiferous tubules of mice.

## 2. Materials and Methods

### Treatments of animals

This experimental study was conducted on 20 adult BALB/C male mice weighing 25-30 gr. The animals were obtained from the animal house of the Faculty of Medicine of Mashhad University of Medical Sciences, Mashhad, Iran, and kept under standard animal housing conditions (12 hr light-12 hr dark cycle at a temperature range of 22-24°C and humidity of 50-55%). During the experiment, the mice had enough access to food and water. All protocol of immunohistochemistry and real-time PCR were carried out at a specialized laboratory of histochemistry and Buali (Avicenna) at the Research Institute of Mashhad University of Medical Sciences.

After the adaptation to the environment, the mice were randomly divided into two groups of hyperthyroidism and control. The control did not receive any interventions, while the hyperthyroidism group was administered with 500 mg/l levothyroxine (L-thyroxine) liquid solution for two 2 months (18). After the treatment period, the hyperthyroidism was confirmed by means of radioimmunoassay after three days in laboratory. Then the mice were kept in the normal conditions of the animal house. After four days, all mice were sacrificed, and their testes were extracted and fixed in formalin. Based on the common histological methods, the samples were molded in paraffin.

### Epididymal smear preparation

For supplying smear, epididymis were removed and divided into small pieces and incubated in 1 ml normal saline, 5% CO${}_{2}$ at 37°C for 30 min. A drop of sperm suspension was placed on a poly-L-lysine lam and fixed for *CATSPER1* and *CATSPER2* immunohistochemical investigation.

### Immunohistochemistry

The animals were sacrificed through cervical dislocation under anesthesia induced with chloroform; subsequently, the epididymis was removed, followed by the incubation of the epididymis fragments mixed with physiological serum at 370°C for 30 min. Next, 10 μl of the suspension containing sperm was spread on a poly-L-lysine slide and then fixed with methanol. For immunohistochemical study, the smears were washed with phosphate-buffered saline (PBS) thrice for 15 min. Subsequently, a standard method was applied for performing heat-induced antigen retrieval. After placing the samples in EDTA/PBS solution at 100°C for 30 min, they were washed in PBS for 15 min. In order to control the peroxidase activity, the samples were immersed in 3% oxygen water, in addition to methanol in darkness for 20 min. Then, they were incubated with PBS for 15 min and 1.5% bovine serum albumin buffer for 30 min at room temperature. Next, the primary *CATSPER1* or *CATSPER2* antibodies were deposited on the samples at a concentration of 1:50 and then incubated (overnight) at 40° for 24 hr. After washing the samples with PBS for 15 min, they were incubated with the donkey anti-goat polyclonal secondary antibody at a concentration of 1: 200 at 37°C for 2 hr. The samples were washed again with PBS, and then placed in a solution containing 0.3 g of 3,3'-diaminobenzidine, 200 μl water, oxygenated water, and PBS for 15 min. After washing the specimens with running water for 1.5 min, they were rinsed with water for 12 min. The samples were then digested using ascending alcohol series (700-1000). Finally, they were sealed for clarification in xylene and between the slide and coverslip. To study the brown color intensity of the samples, an optical microscope (BX51, Olympus, Japan) was connected to a camera with a 100× magnification. The images were reviewed by two researchers in a double-blind manner. The color intensity of the samples in different groups was categorized semiquantitatively (Table I). As indicated in Table I, the samples were analyzed for anti-*CATSPER1* or anti-*CATSPER2* considering light to dark brown staining based on their immunohistochemically staining intensity.

### Messenger RNA expression of *CATSPER1* and *CATSPER2* genes

Total RNA was isolated from the testicular samples by means of the Total RNA Purification kit (Parstous Biotechnology, Iran) following the manufacturer's instructions. The integrity and purity of the obtained RNA were evaluated using electrophoresis on 1.5% agarose gel (Green Viewer, Betagen) and visualized by an ultraviolet transilluminator. The synthesis of the first-strand complementary DNA (cDNA) of the extracted messenger RNA (mRNA) was performed using the Easy cDNA Synthesis kit in the presence of Oligo d (T)${}_{16}$ primer (Parstous Biotechnology, Iran). The mRNA transcripts of *CATSPER1* and *CATSPER2* were examined by the SYBR Green quantitative real-time polymerase chain reaction kit (Parstous Biotechnology, Iran) using the mouse glyceraldehyde-3-phosphate dehydrogenase (GAPDH) gene as the housekeeping gene.

Table II lists the primers applied in the present research. Each reaction (10 µl) contained 5 µl SYBR 2X Mater Mix and 0.5 µM of each specific primer. Amplification was performed with an initial denaturation at 95°C for 10 min, followed by 45 cycles of amplification at 94°C for 20 sec and 60°C for 30 sec using the Rotor-Gene Q (Qiagen, Germany). The reaction mixtures were warmed up at 72°C for 3 min; subsequently, the temperature was ramped to 99°C to analyze the melting curves. To evaluate the gene expression, the cycle thresholds (Ct) for *CATSPER1* and *CATSPER2* genes were normalized to the Ct of the housekeeping gene (i.e., GAPDH). The relative quantification value of the target is expressed as follows:

2-ΔΔ Ct , where ΔCt = Ct (specific gene) - Ct (GAPDH) and ΔΔCt = ΔCt (sample) - ΔCt (calibrator)

ΔCt = CT (target sample) - CT (Referenses)

ΔΔCt = ΔCT (target sample) - ΔCT (control sample)

Ratio= 2-ΔΔ Ct 

**Table 1:** The grade of Immunoreactivity and intensity of reaction for Collagen IV & Lamininα5 Antibody${}^{*}$


**Reaction**	**Grade**
**Negative (-)**	0
**Weak (+)**	1
**Moderate (++)**	2
**Strong (+++)**	3
**Very strong (++++)**	4
${}^{*}$(-), no reaction; (+), weak reaction: light brown; (++), moderate reaction: brown; (+++), strong reaction: dark brown; and (++++), very strong reaction: very dark brown	

**Table 2:** The sequences of primers used in this study


**Gene**	**Primer sequence (5'→3')**
	Forward	TTTACCTGCCTCTTCCTCTTCT
*Catsper1*	Reverse	ACCAGGTTGAGGAAGATGAAGT
		Forward	GGGTGCTGAGGTCTCTCAAAC
*Catsper2*	Reverse	ACCAATGATCCAAGGTGAAGA
		Forward	AACTCCCATTCTTCCACCTTTG
GAPDH${}^{*}$	Reverse	CTGTAGCCATATTCATTGTCATACCA
	${}^{*}$GAPDH, Glyceraldehyde 3-phosphate dehydrogenase		

### Ethical consideration

The animals were used in accordance with the standards of the Animal Research Ethics Committee and accepted by the Ethics Committees of Mashhad University of Medical Sciences (No: IR.MUMS.fm.REC.1394.306).

### Statistical analysis

After final aggregation and accurate recording of all obtained figures, the obtained data were analyzed by using SPSS software version 16 (SPSS Inc., Chicago, Illinois, USA). According to this software, The Mann-Whitney test was used for immunohistochemically reactions and *t* test was used for gene expression in RT-PCR. investigations. The data on average Standard deviation (Mean ± SD) was described. A P-value of less than 0.05 was considered as statistical significant.

## 3. Results

**Figure 1:**
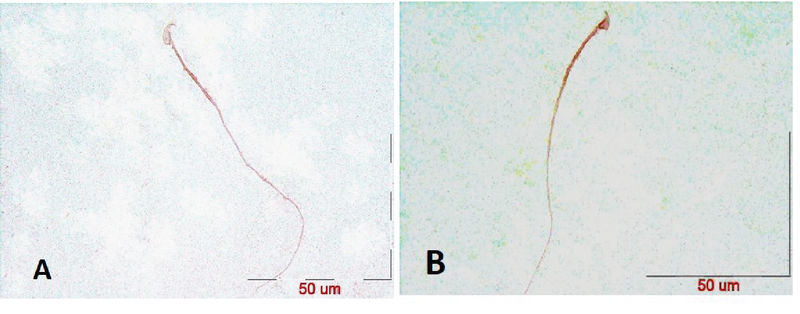
Staining intensity of *CATSPER1* protein by immunohistochemistry in the head, middle piece, and tail of sperm in hyperthyroidism (A) and control (B) groups.

**Figure 2:**
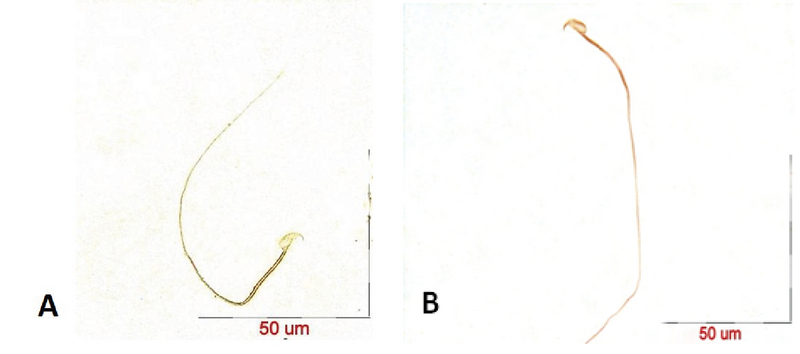
Staining intensity of *CATSPER2* protein by immunohistochemistry in the head, middle piece, and tail of the sperms in hyperthyroidism (A) and control (B) groups.

**Figure 3:**
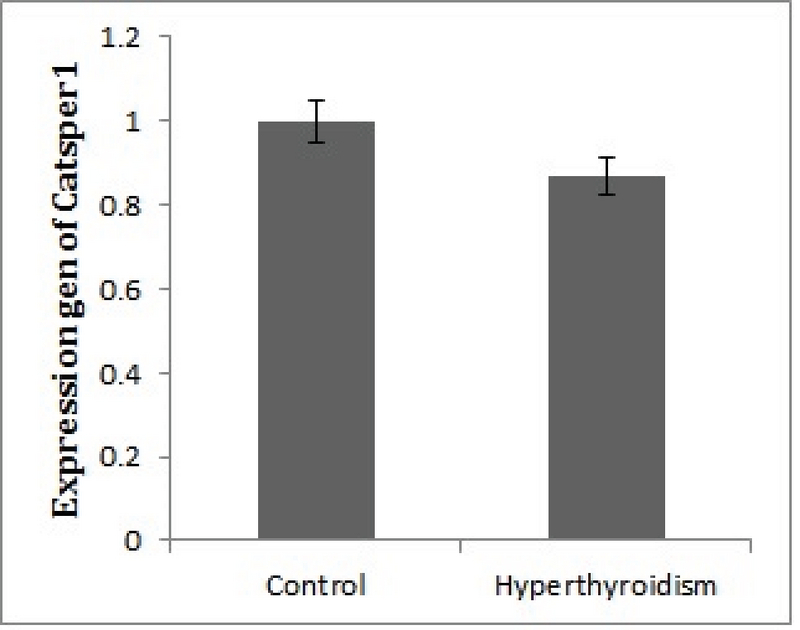
Comparison of *CATSPER1* mRNA expression between the hyperthyroid and control groups. There was no significant difference between the hyperthyroid and control groups (p = 0.45).

**Figure 4:**
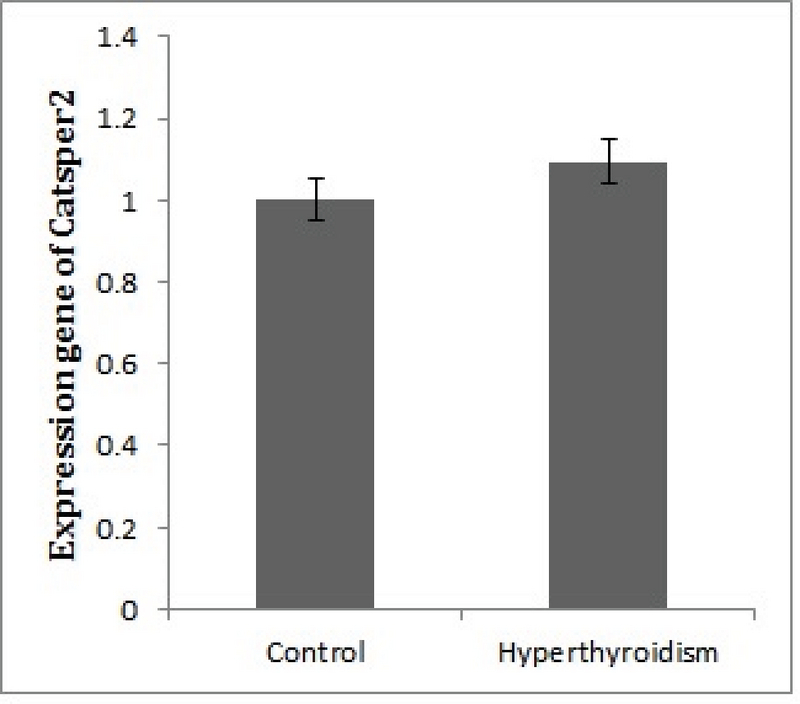
Comparison of *CATSPER2* mRNA expression between the hyperthyroidism and control groups. There was no significant difference between the hyperthyroid and control groups (p = 0.34).

After the confirmation of hyperthyroidism in the experimental mice by the relevant tests, the intensity of immunohistochemical staining was determined in the three parts of the sperm (i.e., head, middle piece, and tail) for both hyperthyroidism and control groups. With regard to *CATSPER1*, there was no reaction in all the three segments of the spermatozoa in the hyperthyroidism group. However, this intensity of coloration was clearly observed in all the three parts of spermatozoa in the control group. In this regard, the head and middle pieces showed high reactions, and the tail demonstrated a moderate reaction (Figure 1). Considering *CATSPER2* in the hyperthyroidism group, the severity of the immunohistochemical staining of the head, middle piece of the sperms was moderate, while that of the tail of the spermatozoa was obtained as low. In the control group, the head and middle piece showed high reactions, while the tail demonstrated a moderate reaction. This comparison showed variation in color from bright to dark brown (Figure 2). The results of the Mann-Whitney U-test revealed no significant differences between the hyperthyroidism and control groups in terms of the severity of immunohistochemical reactions in both *CATSPER1 *(p = 0.17) and *CATSPER2 *(p = 0.22). Similarly, the results of the *t* test also showed no significant difference between the hyperthyroidism and control groups regarding the real-time PCR results of the mRNA gene expression in both *CATSPER1* (p = 0.45; Figure 3) and *CATSPER2* (p = 0.34; Figure 4).

## 4. Discussion

According to the previous study, hyperthyroidism leads to the reduction of some parameters in the Sertoli and Leydig cells in the testes and cellular function in the spermatogenesis process (19). Considering the significant role of *CATSPER* in the molecular and functional structure of the sperm and its motility (11), the present study was targeted toward the investigation of changes in *CATSPER* gene and protein in hyperthyroidism cases. In our study, not only the expression rates of both *CATSPER1* and *CATSPER2 *genes were not significantly different between the hyperthyroidism and control groups, but also the immunohistochemically reactions of anti- *CATSPER1* and *CATSPER2 *antibodies between the two groups. Western blot analysis indicates that the expression level of the *CATSPER1* gene is directly related to sperm with progressive motility. In addition, the expression of this gene in sperm with normal seminal fluid environmental conditions increased as compared to sperm that do not have these Optima conditions (20).

Meanwhile, the expression of *CATSPER1* has a direct relationship with the sperm speed and quality and an inverse relationship with aging. Accordingly, the dead sperms have a low percentage of *CATSPER1* expression (21). In a couple of studies performed by Mohammedi and colleagues on male mice treated with cadmium and nickel, there was a significant difference between the experimental and control groups regarding the expressions of *CATSPER1* and *CATSPER2 *genes, which is not similar to our results. Aside from the toxic effects of selenium on most cells and tissues, it seems that it also has its undesirable effects on *CATSPER1,2* gene and protein expression (22, 23). In another study on adult male mice treated with L-carnitine, the expression rate of *CATSPER1* protein and immunohistochemical reaction of the sperm (head, middle piece, and tail) increased in the experimental group compared to those in the control group. They also obtained similar results regarding *CATSPER2* protein. On the contrary, our results showed a reduction in staining intensity in the hyperthyroidism mice compared to that in the control group (24). Previous findings showed that in hypothyroid mice, hypothyroidism has an effect on the expression *CATSPER1,2* gene and protein that results in significant difference between the experimental group and the control group (25). However, the results of this study on the effects of hyperthyroidism on the aforementioned factors did not show any significant differences in the experimental group compared to the control group. Therefore, it can be concluded that the decrease in thyroid hormone levels may affect the *CATSPER1,2* gene and protein expression (25). In varicocele disease, sgnificant increase was observed in the *CATSPER1,2* gene and protein expression in the experimental group compared to the control group, which did not match with the results of our research in terms of significance. In this regard, study on varicocele was associated with increased testicular temperature. So, increasing temperature may also, in addition to destructive effect on testicular tissue, effect on increasing significant difference on *CATSPER1,2* gene and protein expression (26).

### Limitations 

Blood collection from mice was challenging due to their low blood volume; thus, we used gel tubes provided by the laboratory and performed the required evaluations.

## 5. Conclusion

As the findings of the present study indicated, hyperthyroidism has no significant changes in the expressions of both *CATSPER1* and *CATSPER2 *genes. In addition, no reaction was observed in the histochemical staining intensity of *CATSPER1*. Regarding the *CATSPER2*, the staining intensity in hyperthyroid specimens was weak.

##  Conflict of Interest 

There is no conflict of interests.

## References

[1] Singh R, Hamada AJ, Agarwal A. Thyroid hormones in male reproduction and fertility. *Open Reprod Sci J* 2011; 3: 98–104.

[2] Brent GA. Mechanisms of thyroid hormone action. *J Clin Invest* 2012; 122: 3035–3043. 10.1172/JCI60047 PMC3433956 22945636

[3] Wagner MS, Wajner SM, Maia AL. The role of thyroid hormone in testicular development and function. *J Endocrinol *2008; 199: 351–365. 10.1677/JOE-08-0218 PMC2799043 18728126

[4] Ai J, Zarifkar AA, Takhshid MA, Alavi J, Moradzadeh M. The effect of thyroid activity on adult rat spermatogenesis. *Iran J Vet Res* 2007; 8: 155–160.

[5] Souhila DS, Zohra HS, Kame A, Hadj-Bekkouche F. Effects of thyroxine treatment during lactation on the testicular function of rats across different ages. *Folia Histochem Cytobiol* 2013; 51: 107–114. 10.5603/FHC.2013.0017 23907939

[6] Holsberger DR, Kiesewetter SE, Cooke PS. Regulation of neonatal Sertoli cell development by thyroid hormone receptor α1. *Biol Reprod *2005; 73: 396–403. 10.1095/biolreprod.105.041426 15858214

[7] Liu CR, Li LY, Shi F, Zang XY, Liu YM, Sun Y, et al. Effects of hyper-and hypothyroid on expression of thyroid hormone receptor mRNA in rat myocardium. *J Endocrinol* 2007; 195: 429–438. 10.1677/JOE-07-0253 18000305

[8] Silveira GF, Buffon A, Bruno AN. New approaches to thyroid hormones and purinergic signaling. *J Thyroid Res* 2013; 2013: 434727. 10.1155/2013/434727 PMC3730180 23956925

[9] Li HG, Ding XF, Liao AH, Kong XB, Xiong CL. Expression of CatSper family transcripts in the mouse testis during post-natal development and human ejaculated spermatozoa: relationship to sperm motility. *Mol Hum Reprod *2007; 13: 299–306. 10.1093/molehr/gam009 17347248

[10] Park EH, Kim DR, Kim HY, Park SK, Chang MS. Panax ginseng induces the expression of CatSper genes and sperm hyperactivation. *Asian J Androl* 2014; 16: 845–851. 10.4103/1008-682X.129129 PMC4236327 24969054

[11] Singh AP, Rajender S. CatSper channel, sperm function and male fertility. *Reprod Biomed Online *2015; 30: 28–38. 10.1016/j.rbmo.2014.09.014 25457194

[12] Xia J, Reigada D, Mitchell CH, Ren D. CATSPER channel-mediated Ca2+ entry into mouse sperm triggers a tail-to-head propagation. *Biol Reprod* 2007; 77: 551–559. 10.1095/biolreprod.107.061358 17554080

[13] Amini M, Shirinbayan P, Behnam B, Roghani M, Farhoudian A, Joghataei MT, et al. Correlation between expression of CatSper family and sperm profiles in the adult mouse testis following I ranian Kerack abuse. *Andrology* 2014; 2: 386–393. 10.1111/j.2047-2927.2014.00195.x 24619711

[14] Mohammadi S, Jalali M, Nikravesh MR, Fazel A, Ebrahimzadeh A, Gholamin M, et al. Effects of Vitamin-E treatment on CatSper genes expression and sperm quality in the testis of the aging mouse. *Iran J Reprod Med *2013; 11: 989–998. PMC3941406 24639725

[15] Qi H, Moran MM, Navarro B, Chong JA, Krapivinsky G, Krapivinsky L, et al. All four CatSper ion channel proteins are required for male fertility and sperm cell hyperactivated motility. *Proc Nati Acad Sci USA *2007; 104: 1219–1223. 10.1073/pnas.0610286104 PMC1770895 17227845

[16] Loux SC, Crawford KR, Ing NH, González-Fernández L, Macías-García B, Love CC, et al. CatSper and the relationship of hyperactivated motility to intracellular calcium and pH kinetics in equine sperm. *Biol Reprod* 2013; 89: 123–137. 10.1095/biolreprod.113.111708 24048572

[17] Ren D, Navarro B, Perez G, Jackson AC, Hsu S, Shi Q, et al. A sperm ion channel required for sperm motility and male fertility. *Nature* 2001; 413: 603–609. 10.1038/35098027 PMC8462998 11595941

[18] Ferreira E, Silva AE, Serakides R, Gomes AES, Cassali GD. Model of induction of thyroid dysfunctions in adult female mice. *Arq Bras Med Vet Zootec* 2007; 59: 1245–1249.

[19] Rijntjes E, Wientjes AT, Swarts HJ, de Rooij DG, Teerds KJ. Dietary-induced hyperthyroidism marginally affects neonatal testicular development. *J Androl* 2008; 29: 643–653. 10.2164/jandrol.108.005108 18599886

[20] Tamburrino L, Marchiani S, Minetti F, Forti G, Muratori M, Baldi E. The CatSper calcium channel in human sperm: relation with motility and involvement in progesterone-induced acrosome reaction. *Hum Reprod *2014; 29: 418–428. 10.1093/humrep/det454 24430778

[21] Tamburrino L, Marchiani S, Vicini E, Muciaccia B, Cambi M, Pellegrini S, et al. Quantification of CatSper1 expression in human spermatozoa and relation to functional parameters. *Hum Reprod *2015; 30: 1532–1544. 10.1093/humrep/dev103 25983333

[22] Mohammadi S, Gholamin M, Mansouri A, Mahmoodian RS, Babazadeh B, Kebriaei SM, et al. Effect of cadmium and nickel on expression of CatSper 1 and 2 genes in mice. *Toxin Rev* 2018; 37: 216–222.

[23] Mohammadi S, Movahedin M, Mowla SJ. Up-regulation of CatSper genes family by selenium. *Reprod Biol Endocrinol *2009; 7: 126–131. 10.1186/1477-7827-7-126 PMC2780429 19917098

[24] Mohammadi S, Jalali M, Nikravesh MR, Gholamin M, Fazel A, Ebrahimzadeh A, et al. Effects of L-carnitine treatment on expression of CatSper proteins in the aging mouse model. *Eur J Exp Biol* 2013; 3: 731–735.

[25] Alipour F, Jalali M, Nikravesh MR, Fazel A, Sankian M, Khordad E. Assessment of sperm morphology, chromatin integrity, and catSper genes expression in hypothyroid mice. *Acta Biol Hung* 2018; 69: 244–258. 10.1556/018.68.2018.3.2 30257580

[26] Soleimani MZ, Jalali Mashayekhi F, Mousavi Hasanzade M, Baazm M. Alteration in CatSper1 and 2 genes expression, sperm parameters and testis histology in varicocelized rats. *Int J Reprod Biomed* 2018; 16: 183–190. PMC5944440 29766149

